# Retraction: Serum Magnesium in Patients With Acute Myocardial Infarction and Its Effect on Cardiac Complications and Mortality in Myocardial Infarction Patients

**DOI:** 10.7759/cureus.r181

**Published:** 2025-04-30

**Authors:** Zia Sabah, Javed Wani, Mosab Deajim, Ahmed S AL Zomia, Abdullah Asiri, Ali A Alqahtani, Wasna Alansari, Amin Alwan, Ryan M AL Qahtani, Berin Raj

**Affiliations:** 1 Medicine, King Khalid University, Abha, SAU; 2 Internal Medicine, King Khalid University, Abha, SAU; 3 Medicine and Surgery, King Khalid University, Abha, SAU; 4 Medicine, King Saud Bin Abdulaziz University for Health Sciences, Jeddah, SAU; 5 Family and Community Medicine, King Fahad General Hospital, Gizan, SAU; 6 Epidemiology and Public Health, Prince Faisal Bin Khalid Cardiac Center, Abha, SAU

The editors-in-chief have retracted this article due to significant discrepancies between the study’s stated objectives and the data presented. While the article aimed to evaluate the effect of serum magnesium levels on cardiac complications and mortality in acute myocardial infarction patients, no mortality data were collected, analyzed, or reported. This omission fundamentally misrepresents the scope and findings of the study. Additionally, the absence of multivariate analysis and the overinterpretation of observational data as having clinical applicability raise concerns about the validity and reliability of the conclusions. The authors do not agree with the decision to retract.

